# Recovery of Bioactive Compounds from the Biomass of Aromatic Plants After Distillation Using NADES: A Sustainable Alternative Extraction Method

**DOI:** 10.3390/molecules30051120

**Published:** 2025-02-28

**Authors:** Eleonora Truzzi, Davide Bertelli, Benedetta Catellani, Danial Darvishi Jazi, Stefania Benvenuti

**Affiliations:** Department of Life Sciences, University of Modena and Reggio Emilia, Via G. Campi 103, 41125 Modena, Italy; davide.bertelli@unimore.it (D.B.); benedetta.catellani@unimore.it (B.C.); 225966@studenti.unimore.it (D.D.J.)

**Keywords:** NADES, *Lamiaceae*, *Asteraceae*, distillation, biomass, essential oils, HPLC-DAD, UHPLC-HRMS, GC-FID, GC-MS, polyphenols

## Abstract

The extraction processes for medicinal plants, particularly the distillation of aromatic plants, generate significant quantities of by-products, consisting of fibrous biomass and hydrosols. These by-products pose challenges for disposal and recovery. Consequently, it is imperative to make the entire highly energy-intensive process more sustainable by valorizing all derivatives. This study aims to recover polyphenols from the exhausted biomasses of *Artemisia dracunculus*, *Echinacea purpurea*, *Helichrysum italicum* (from the *Asteraceae* family), and *Lavandula angustifolia*, *Lavandula × intermedia*, *Melissa officinalis*, *Salvia officinalis*, *Salvia sclarea*, and *Salvia rosmarinus* (from the *Lamiaceae* family) after steam distillation. The residual biomasses were extracted using ethanol (conventional solvent) and different natural deep eutectic solvents (NADES) composed of choline chloride in combination with citric and lactic acids at different molar ratios. The NADES containing choline chloride and lactic acid at the molar ratio 1:1 (CLA11) exhibited the highest recovery of representative phenols of the plants, namely chicoric and rosmarinic acids. The CLA11 solvent demonstrated a stronger extractive capacity compared to ethanol in all the biomasses belonging to the *Asteraceae* and *Lamiaceae* families. Specifically, CLA11 extracts showed a higher number of compounds in UHPLC-HRMS and greater concentrations of chicoric and rosmarinic acids determined by HPLC-DAD than ethanol extracts. In conclusion, NADES were demonstrated to be a viable alternative system for the recovery of bioactive compounds that could be used to formulate new products for the food, pharmaceutical, and cosmetic industries. Moreover, the use of NADES can enhance the sustainability of the whole production chain of essential oils being environmentally friendly.

## 1. Introduction

Aromatic plants have always been used in the therapeutic field due to the abundance of bioactive compounds contained in essential oil (EO). Currently, aromatic plants find employment in the pharmaceutic, cosmetic, food, and agriculture industries [1]. The main bioactive compounds in aromatic plants are terpenes and terpenoids, constituents of EOs, known for their antiseptic activity, medicinal properties, and fragrance. They are used as antimicrobial agents [2,3], analgesics [4], sedatives [4], anti-inflammatories [5], spasmolytics, local anesthetics, and anti-cancer agents [6,7,8]. EOs, as described by European Pharmacopeia, are volatile mixtures of odorous compounds, usually of complex composition, obtained from a botanically defined herbal drug by steam distillation, dry distillation, or a suitable mechanical process without heating, from roots, leaves, flowers, and fruit peels of aromatic plants. The extraction processes of EOs from aromatic plants by means of industrial techniques generate significant quantities of by-products, consisting of fibrous biomass and hydrosols. Moreover, extracting EOs from aromatic plants by steam distillation is a highly expensive process that requires consistent water and energy usage. Therefore, the valorization of the wastes is extremely important to make the production of EOs more environmentally sustainable and help accomplish the new circular economy action plan, one of the main blocks of the European Green Deal.

Currently, the exhausted biomasses are mainly employed for producing biofuel; however, this is the less preferable procedure to manage biomass, according to the “waste hierarchy” proposed by the Environmental Protection Agency (EPA). According to the EPA’s scheme, waste reuse and recycling are favored over energy recovery. These agri-food wastes can be exploited for more noble purposes, being rich in bioactive compounds such as polyphenols, before the production of biofuel [9,10]. Polyphenols are a large class of chemical compounds that include phenolic acids, flavonoids, anthocyanins, proanthocyanins, and stilbenes with marked health benefits. Several studies have recently demonstrated the countless potentialities of polyphenols as therapeutic agents and food preservatives due to their anti-inflammatory, antimicrobial, antioxidant, and enzyme-inhibitory activities [11].

These compounds are usually extracted via conventional methods that employ inflammable, toxic, and contaminant organic solvents. In recent years, research efforts have focused on the development of more sustainable strategies, using green technologies with higher process performances and solvents with a lower environmental impact. In this context, natural deep eutectic solvents (NADES) have been proposed as safe and environmentally friendly alternatives to classic solvents. The NADES rapidly showed potential in green chemistry due to their low cost, recyclability, biodegradability, biocompatibility, and non-toxicity. The NADES are eutectic mixtures composed of a hydrogen bond acceptor (HBA) and a hydrogen bond donor (HBD) that create a dense bend network due to hydrogen bonding and van der Waals interactions [12]. Therefore, NADES blends are capable of efficiently solubilizing lipophilic compounds and protecting thermolabile compounds. The efficiency and significance of NADES as solvents are attributed to the fact that these mixtures are inside cells. Indeed, when combined in appropriate ratios, mixtures of numerous primary metabolites can form natural deep eutectic solvents. The presence of NADES inside cells is crucial because many macromolecules, such as DNA, proteins, and polysaccharides, which are poorly soluble in aqueous phase, can dissolve within these mixtures. Eutectic bends high solubilizing capacity is related to their molecular structure and wide polarity range. NADES can also play an important role in safeguarding organisms from harsh conditions, such as drought and cold [13]. In the last years, several studies have successfully demonstrated the potentiality of NADES in extracting polyphenols from aromatic plants [14,15,16,17]. The employment of NADES in the recovery of bioactive compounds of aromatic plant by-products has not been considered so far.

For these reasons, the project aims at the use of NADES for the extraction of polyphenols and other bioactive compounds from the exhausted biomasses of several aromatic plants which are considered agri-food wastes. To the best of our knowledge, this is the first study applied to the by-products from the steam distillation of aromatic plants. The research also aims to develop a novel and environmentally sustainable approach to valorize the by-products from the production of EOs that can be exploited in different fields. Indeed, since NADES are totally biocompatible and non-toxic for humans, animals, and the environment, it is important to further investigate their formulation in foods and feeds enriched with polyphenols and other bioactive compounds. Moreover, NADES could be interestingly proposed as a substitute for conventional extractive solvents that are inflammable and harmful to the environment.

In this regard, the NADES were considered for the extraction of polyphenolic compounds from the biomasses of several *Lamiaceae* and *Asteraceae* plants widely distributed and cropped in the north of Italy for their characteristic aroma and therapeutic properties. Specifically, *Artemisia dracunculus* L. (ART), *Echinacea purpurea* (L.) Moench (ECHI), *Helichrysum italicum* (Roth) G. Don (HEL), *Lavandula angustifolia* Mill. (LAV), *Lavandula × intermedia* Emeric ex Loisel (LAI), *Melissa officinalis* L. (MEL), *Salvia officinalis* L. (SAO), *Salvia rosmarinus* Spenn. (ROS), and *Salvia sclarea* L. (SAS) were selected. Different NADES formulations were prepared and their extraction capability was compared to that of ethanol, a commonly extractive organic solvent. The chemical characterization of the EOs was made using gas chromatography coupled with a mass spectrometer (GC-MS) and flame ionization (FID) detectors. The extraction capacity of NADES compared to traditional solvents was studied by Ultrahigh-Performance Liquid Chromatography–High-Resolution Mass Spectrometry (UHPLC-HRMS) to determine the polyphenolic composition, and High-Performance Liquid Chromatography with Diode Array Detection (HPLC-DAD) to quantify the characteristic active components of the plants belonging to the *Asteraceae* and *Lamiaceae* families.

## 2. Results

### 2.1. Chemical Characterization of the EOs

The chemical characterization of the EOs obtained from the aerial parts of the aromatic plants was performed using GC–MS and GC-FID analysis. Table 1 summarizes the relative peak areas of each component, elution order, and comparison between experimental (exp) and literature (lit) linear retention index (LRI) values.

Overall, the chemical composition of aromatic plants was extremely variable mainly due to both the different family and genus. A less marked variability was ascribable to the species of the plants under investigation. The differences in EO composition are imputable to the individual genetic variability of plants that influences the biosynthetic pathways of secondary metabolites. Other factors that can impact the chemical composition of the EOs are the variation among different plant parts, time of harvesting, and pedoclimatic conditions of plant growth [18]. In this study, these latter factors did not affect the differences in the chemical composition of EOs within the aromatic plants. Indeed, all plants were collected at the balsamic time and the aerial parts, the plant part richest in EOs, were steam-distilled. Also, the pedoclimatic conditions influence the variability of the EOs obtained from the same species and cultivar and not that of EOs with completely different genetics.

The EOs belonging to the *Lamiaceae* family displayed a high content of oxygenated monoterpenes among which alcohols, aldehydes, ketones, and esters depending on the genus. *Lavandula × intermedia* and *Lavandula angustifolia* were mainly characterized by linalool (20.19% and 35.19%, respectively) and its ester linalyl acetate (36.63% and 34.01%, respectively). Also, both the EOs contained 1,8-cineole, cis-β-ocimene, trans-β-ocimene, fenchol, camphor, borneol, terpinen-4-ol, lavandulyl-acetate, β-caryophyllene, and β-caryophyllene-oxide in characteristic and specific quantities that indicate a different biosynthetic pathway of the two species [19]. *Melissa officinalis* was characterized by high concentrations of aldehydes (citronellal 9.43%, geranial 12.74%, and neral 6.49) and caryophyllene derivatives β-caryophyllene (17.08%) and caryophyllene-oxide (20.64%) [20].

The EO of *Salvia rosmarinus* showed a pinene chemotype due to the high concentration of the hydrocarbon monoterpene (α-pinene 38,79%), according to [21]. Significant percentages of 1,8-cineole (18.55%) and verbenone (6.04%) were detected in agreement with the literature [22].

Finally, the sage EOs displayed an extremely different chemical composition. Specifically, *Salvia officinalis* was represented by 38% of hydrocarbon monoterpenes, and the ketones camphor and α- and β-thujone which accounted for almost 38% of the total composition. Conversely, *Salvia sclarea* exhibited a chemical composition close to that of *Lavandula angustifolia*, being mainly characterized by linalool (15.87%) and its ester linalyl acetate (70.96%) [23].

Regarding the EOs extracted from the aerial parts of the *Asteraceae* plants as indicated by GC–MS characterization and GC-FID quantification, ART EO consisted of high amount of the phenylpropanoid estragol (65.15%), confirming the results of previous investigations [24]. Moreover, this oil was also characterized by monoterpene hydrocarbons, such as cis-ß-ocimene (11.50%), trans-ß-ocimene (15.70%). In ECHI EO, α-pinene, β-pinene, myrcene, and p-cymene were the most concentrated monoterpenes accounting for 15.76% of the total composition. Conversely, germacrene D was the most abundant hydrocarbon sesquiterpene, representing 66.43% of the total composition [25]. Concerning HEL EO, the most concentrated compounds were α-pinene, ar-curcumene, italidione II, limonene, β-caryophyllene, italicene, and α-selinene [26].

**Table 1:** Semiquantitative chemical composition (%) of the essential oils obtained by steam distillation from *Lavandula × intermedia* (LAI), *Lavandula angustifolia* (LAV), *Melissa officinalis* (MEL)*, Salvia rosmarinus* (ROS)*, Salvia officinalis* (SAO), *Salvia sclarea* (SAS), *Artemisia dracunculus* (ART), *Echinacea purpurea* (ECHI)*,* and *Helichrysum italicum* (HEL).

Compound	LRI _exp_	LRI _lit_	LAI	LAV	MEL	ROS	SAO	SAS	ART	ECHI	HEL
**α-thujene**	928	928	-	-	-	0.25	0.19	-	-	-	-
**α-pinene**	935	936	0.28	0.60	0.18	38.79	3.84	-	1.53	5.56	24.53
**Camphene**	943	950	0.49	0.18	-	5.37	6.29	-	0.13	0.17	0.99
**Sabinene**	969	973	0.25	0.42	-	2.58	4.39	-	-	-	0.15
**β-pinene**	971	975	-	0.15	1.05	-	0.12	-	0.17	1.33	1.07
**Octanone**	988	985	0.61	0.43	-	0.62	-	-	-	-	0.17
**Myrcene**	989	992	0.97	1.21	0.54	2.55	2.00	1.40	0.15	7.98	-
**α-phellandrene**	1001	1005	-	-	-	0.30	-	-	-	0.76	-
**δ-3-carene**	1007	1010	0.12	0.48	-	-	-	-	-	-	-
**α-terpinene**	1017	1017	0.41	0.18	-	0.56	-	-	-	-	0.24
***p*** **-cymene**	1024	1026	0.67	0.29	0.16	0.40	0.40	0.23	-	0.89	0.29
**Limonene**	1029	1030	0.43	1.07	-	-	21.42	-	3.63	-	7.77
**1,8-cineole**	1031	1032	6.16	1.62	-	18.55	-	-	-	-	0.76
***cis*** **-β-ocimene**	1037	1039	0.49	3.04	-	-	-	0.37	11.50	-	-
***trans*** **-β-ocimene**	1049	1049	0.26	4.04	-	-	-	0.63	15.70	-	0.32
**γ-terpinene**	1058	1061	0.11	0.13	-	1.12	-	-	-	-	0.71
**Terpinolene**	1085	1088	-	-	-	0.91	0.11	0.11	-	-	0.21
**Linalool**	1098	1102	20.19	35.19	0.61	1.82	0.15	15.87	-	-	0.87
**α-thujone**	1104	1101	-	-	-	-	10.70	-	-	-	-
**6-camphenol**	1105	1106	0.73	-	0.21	0.26	-	-	-	-	-
**α-fenchol**	1114	1116	2.22	1.04	0.25	-	-	-	-	-	-
**β-thujone**	1115	1112	-	-	-	-	5.70	-	-	-	-
***Trans*** **-rose oxide**	1126	1128	0.20	-	-	0.31	-	-	0.24	-	-
**Camphor**	1144	1145	3.52	0.35	-	3.83	22.41	-	-	-	-
**Nerol oxide**	1153	1148	-	-	-	-	-	-	-	-	0.72
**Citronellal**	1155	1157	-	-	9.43	0.17	-	-	-	-	-
**Menthone**	1157	1155	-	-	0.22	1.97	-	-	-	-	-
**Borneol**	1166	1168	1.80	1.32	-	3.79	1.62	-	-	-	-
**Lavandulol**	1169	1172	0.25	-	-	0.78	-	-	-	-	-
**Terpinen-4-ol**	1175	1181	1.01	5.59	-	0.78	0.43	-	-	-	0.31
***p*** **-cymen-8-ol**	1188	1185	0.24	-	0.76	-	0.19	-	-	-	-
**α-terpineol**	1189	1193	0.34	0.64	-	1.03	-	2.09	-	-	0.35
**Myrtenal**	1194	1195	0.31	0.15	-	0.29	-	-	-	-	-
**Estragole**	1200	1195				-			65.15		
**Verbenone**	1207	1204				6.04					
**Nerol**	1230	1232	-	-	0.18	-	-	0.19	-	-	1.04
**Citronellol**	1233	1233	-	-	0.18	-	-	0.15	-	-	-
**Neral**	1245	1245	0.15	-	6.49	-	-	-	-	-	-
**Piperitone**	1255	1252	0.13	-	0.50	1.23	-	-	-	-	-
**Linalyl acetate**	1264	1259	38.63	34.01	0.30	-	-	70.96	-	-	-
**Geraniol**	1267	1267	0.15	-	2.02	-	-	-	-	-	-
**Geranial**	1274	1269	-	-	12.74	-	-	-	-	-	-
**Bornyl acetate**	1288	1288	0.32	0.80	0.33	1.32	1.50	-	0.11	-	-
**Lavandulyl acetate**	1294	1290	3.13	-	-	-	-	-	-	-	-
**δ-elemene**	1338	1337	0.73	-	0.24	-	-	-	-	0.27	-
**Neryl acetate**	1367	1368	0.21	0.29	-	-	-	0.73	-	-	1.54
**α-copaene**	1377	1376	0.29	-	0.69	-	0.11	0.36	-	0.24	1.31
**Geranyl acetate**	1385	1386	-	-	3.50	-	-	1.43	-	-	-
**β-bourbonene**	1389	1384	-	-	0.41	-	0.11	0.20	-	-	-
**β-cubebene**	1392	unknown	0.44	0.67	0.39	-	-	-	-	0.94	-
**β-elemene**	1395	1390	0.20	-	-	-	-	0.14	-	-	-
**Italicene**	1404	1409	-	-	-	-	-	-	-	-	2.65
**Cis-α-bergamotene**	1416	1415	-	-	-	-	-	-	-	-	0.86
**β-caryophyllene**	1420	1420	1.97	3.30	17.08	2.46	4.62	0.99	0.23	2.50	3.49
**α-humulene**	1431	1433	-	0.34	1.93	-	-	-	-	0.65	-
**Trans-α-bergamotene**	1440	1436	0.12	0.26	-	-	1.80	-	-	-	-
**Neryl propanoate**	1455	1452	-	-	-	-	-	-	-	-	2.47
**β-farnesene**	1456	1457	1.20	-	0.15	-	-	-	-	1.13	0.31
**alloaromadendrene**	1464	1466	-	-	-	0.34	0.23	-	-	-	-
***γ*** **-curcumene**	1478	1480	-	-	-	-	-	-	-	-	2.01
**ar-curcumene**	1483	1484	0.37	0.43	-	-	0.15	-	0.38	-	9.85
**β-selinene**	1485	1489	-	-	-	-	-	-	0.25	-	2.41
**Germacrene D**	1488	1481	-	-	1.10	-	0.12	2.83	-	66.43	-
**Italidione II**	1491	1493	-	-	-	-	-	-	-	-	9.55
**α-selinene**	1499	1496	-	-	-	-	0.75	0.56	-	1.86	6.38
**β-bisabolene**	1510	1511	-	-	-	-	-	-	-	-	0.26
**β-curcumene**	1513	1513	-	-	-	-	-	-	-	-	0.37
**γ-cadinene**	1522	1524	0.12	-	0.41	-	-	-	-	-	0.14
**δ-cadinene**	1526	1523	0.69	0.24	0.19	-	0.28	-	-	1.31	0.11
**Nerolidol**	1559	1563	-	-	0.97	-	-	-	-	-	-
**Spathulenol**	1580	1577	-	-	-	-	0.76	-	-	0.57	-
**Italidione III**	1583	1583	-	-	-	-	-	-	-	-	0.96
**Caryophyllene oxide**	1590	1589	2.35	0.23	20.64	0.14	0.96	0.13	-	1.65	-
**TOTAL**			93.64	98.71	94.04	99.53	92.00	99.37	99.54	96.82	87.01

Experimental retention indices and literature retention indices (HP-5 column) according to NIST [27].

### 2.2. Optimization of NADES Extraction

The oil-exhausted aerial parts biomasses were extracted via ultrasonication using EtOH 70%, as conventional solvent, and the NADES formulations. EtOH 70% was selected for comparison as one of the most employed solvents for the recovery of polyphenols, such as rosmarinic acid, being efficient and generally recognized as safe (GRAS) [28]. Indeed, the employment of EtOH is allowed in the preparation of feed and food products. The percentage of EtOH in the hydroalcoholic solvent can be varied depending on the part of the plant to be extracted and the compound targets [29]. Ultrasound-assisted extraction is considered an environmentally friendly methodology due to the shorter processing time and higher extraction yield compared to dynamic maceration, one of the most employed extraction techniques [30]. The higher recovery of polyphenol is ascribable to the cavitation phenomenon, which induces the formation, growth, and collapse of cavitation bubbles. The cavitation bubbles promote the disruption of the cell walls of the plant material and increase the contact area with the solvent, resulting in a fast release of bioactive compounds. NADES formulations were prepared selecting ChCl as the HBA. ChCl is one of the most popular natural HBA due to its affordability, biodegradability, safety, and health-beneficial effects. Also, the European directive 70/524/EEC8 authorized the employment of ChCl in feeds as an additive without time limitation (without a periodic re-evaluation of the safety) [31]. ChCl was proposed in combination with several organic acids, among which lactic (LA) and citric (CA) acids, to extract polyphenols from foods and aromatic plants [32,33,34,35,36]. LA and CA were selected among the other organic acids for their low toxicity and low costs. Moreover, NADES formulations composed of these organic acids have been reported to efficiently extract polyphenols due to the polarity of the resulting eutectic solvent [37,38]. Since the ultrasonic variables have a strong impact on the extraction performances of NADES, temperature and time of extraction were kept fixed to evaluate only the contribution of the chemical composition of eutectic solvents [30]. The molarity of ChCl and the amount of water were kept fixed and their ratio with LA and CA was varied to modulate to some extent the NADES properties, such as polarity and extraction affinity.

**Figure 1:**
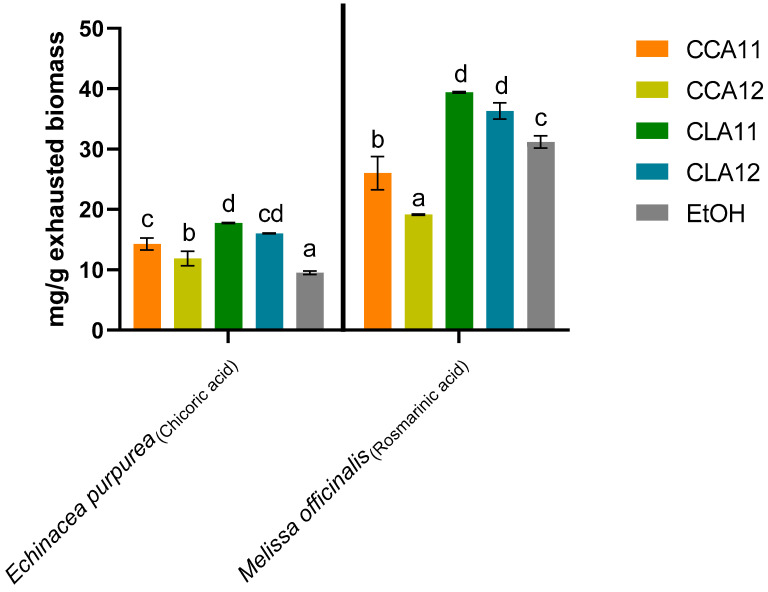
Amount of chicoric and rosmarinic acids extracted with ethanol (EtOH) and NADES formulations in *Echinacea purpurea* and *Melissa officinalis* biomasses. CCA11 and CCA12 are the NADES formulations composed of choline chloride and citric acid at the molar ratio 1:1 and 1:2, respectively; CLA11 and CLA12 are the NADES formulations composed of choline chloride and lactic acid at the molar ratio 1:1 and 1:2, respectively. Distinct letters were used to differentiate statistically different groups according to Tukey’s post hoc test (*p* < 0.05).

The extraction performance of NADES formulations and EtOH was investigated by HPLC-DAD determining the concentration of the most representative phenolic acid. To evaluate which NADES mixture has the highest extraction capacity, the reference samples used were ECHI and MEL for the *Asteraceae* and *Lamiaceae* families, respectively. Specifically, chicoric acid and rosmarinic acid contents within the various were considered, according to the literature [39] (Figure 1).

Overall, the weakest NADES was that composed of ChCl and CA at the molar ratio 1:2 (CCA12) followed by that composed of ChCl and CA at the molar ratio 1:1 (CCA11). In the case of MEL, these eutectic formulations were less efficacious than EtOH in extracting rosmarinic acid. Conversely, in the case of ECHI, EtOH achieved the lowest recovery of chicoric acid. The evidence that EtOH 70% was less efficient in extracting chicoric acid in ECHI than rosmarinic acid in MEL might be ascribable to the chemical structure of the two phenolic acids, as also observed by Oliva et al. on other polyphenols [40]. Indeed, chicoric acid is more polar than rosmarinic acid due to the presence of tartaric acid between the two hydroxycinnamic acids. The lower recovery of chicoric acid by EtOH compared to all NADES in ECHI might be explained by the poor extractability performance of partially ionized compounds by EtOH, as observed by Torres-Vega et al. for the recovery of boldine with MeOH [41]. Han and co-workers also highlighted that the extractive performance of conventional solvents and NADES depends on the chemical structure of each polyphenol. They observed that overall NADES composed of betaine and urea/malic acid at the molar ratio 1:1 recovered higher concentrations of polyphenols than ethanol; however, specific phenols or flavonoids were more concentrated in EtOH extracts than NADES extracts [42].

The extraction of chicoric acid with ChCl and LA at the molar ratio 1:1 (CLA11) was significantly higher than that obtained with the other NADES formulations in ECHI (*p* < 0.05). The same results were obtained for the rosmarinic acid in MEL (*p* < 0.05). A similar result was achieved by Ivanović et al. in *H. arenarium*, where only the NADES CLA11 with 25% of water displayed a higher extractive capacity for dicaffeoylquinic acids (derivative of caffeic acid as chicoric and rosmarinic acids) than MeOH 80% along with ChCl and 1,2-propanediol (molarity ratio 1:2) [43].

The variation of the ratio of LA with the HBA (CLA11 and CLA12) did not significantly affect the extraction of rosmarinic acid in MEL and chicoric acid in ECHI. The greater extractive capacity of LA compared to CA has already been reported in the literature on different natural matrices [30,44,45]. These differences might be related to various factors affecting the extraction capacity, such as viscosity, polarity, pH, and the number of hydrogen bond acceptor and donator groups. LA and CA differ in the number of carboxylic groups and molecular size. The larger molecular size of CA with three carboxylic groups compared to LA ensures a stronger interaction with ChCl, resulting in an increased viscosity of the NADES [46]. Therefore, the presence of high concentrations of CA affects the viscosity of the resulting NADES. Thus, the higher viscosity of CA-based NADES than LA-based NADES could have impaired the extractive performance since the high viscosity of NADES has been reported to hinder the extractive efficiency [47] and decrease the mass transfer and diffusivity of compounds. The number of functional groups also affects the pH of the solvent in addition to the viscosity. Overall, acidic conditions are preferable for the extraction of polyphenols, as these compounds remain in their non-dissociated form at low pH. However, highly acidic NADES with a pH close to 0, such as ChCl/CA, may hinder the interaction of phenols. These factors could also explain why, in our study, the NADES with a 1:1 molar ratio of ChCl to LA (CLA11) exhibited slightly greater extractive strength than CLA12 [48].

### 2.3. Analysis of Selected NADES and EtOH Extracts

All the biomasses were extracted using the NADES formulation CLA11, as it provided the highest recovery of the most characteristic polyphenols. The EtOH and NADES extracts were qualitatively analyzed by UHPLC-HRMS to identify all metabolites (Table 2).

The polyphenolic profile of the extracts was characterized by the presence of compounds from the phenolic acid and flavonoid classes. Overall, the NADES extracts contained a greater number of metabolites than the EtOH extracts, due to the higher extractive power of the eutectic solvent, as also observed by Oliva et al. on strawberry waste [40]. Additionally, the *Lamiaceae* biomasses exhibited a greater diversity of metabolites compared to the *Asteraceae* biomasses.

The phenolic acids were represented by hydroxycinnamic acid derivatives except for the protocatechuic acid hexose (**2**), a dihydroxybenzoic acid. The hydroxycinnamic class was constituted by esters of caffeic, coumaric, and ferulic acids or their derivatives. Compounds **1**, **2**, **3**, **4**, **5**, **8**, **9**, **16**, **17**, **18**, **22**, **24**, **26**, **27**, **28**, **31**, **32**, **33**, **34**, **35**, and **36** were identified as caffeic acid derivatives due to the presence of the characteristic fragment ions at *m*/*z* 179.034, 161.023, and 135.044. Specifically, the parent ion **1** at *m*/*z* 197.045 was assigned to the hydration product of caffeic acid, danshensu (3,4-dihydroxyphenyl lactic acid), due to the loss of the hydroxylic group (18 Da). Metabolites **4** (and **9**) and **5** (and **8**) were recognized as caffeoylquinic acid and caffeoyl hexose, respectively. The fragments ions of the caffeic acid were generated by the neutral loss of the quinic acid (−191 Da) and the hexose (−162 Da) moieties. Similarly, compound **3** was identified as caftaric acid (caffeoyltartaric acid) because of the neutral loss of 132 Da ascribable to the tartaric acid moiety. All the other caffeic acid derivatives were the product of the condensation of caffeic acid with one or more other phenolic acids. The most abundant derivative in the extracts of *Lamiaceae* biomasses was the rosmarinic acid. The peaks **24** and **22** were assigned to rosmarinic acid and its hexoside. Rosmarinic acid was characterized by the precursor ion at *m*/*z* 359.076 that yielded the daughter ions at *m*/*z* 197.044 and 179.034 that correspond to the deprotonated danshensu and caffeic acid, respectively. The hexoside at *m*/*z* 521.034 underwent the loss of hexose moiety (−162 Da) and generated the characteristic fragments of the rosmarinic acid. Compounds **30**, **32**, **33**, **36**, and **37** were recognized as salvianolic acids, other characteristic metabolites of *Lamiaceae* generated from the condensation of hydroxycinnamic acids. Metabolite **37** was found the product of the condensation of two rosmarinic acids. The differences within these metabolites are the ratios between danshensu and caffeic acid [63]. Caffeic acid was also found condensed with a coumarin in peaks **34** and **35**.

The parent ions **16** and **18** at *m/z* 539.119 and 597.125 were assigned to yunnaneic acid D and F, respectively. From the precursor ion **16**, a neutral loss of caffeic acid yielded the fragment at *m*/*z* 359.077 (rosmarinic acid). Yunnaneic acid was putatively identified for the presence of the characteristic fragmentation ions at *m*/*z* 311.093, 197.045, 179.034, and 135.044 [48]. Chicoric acid (dicaffeoyltartaric acid) was assigned to peak **17** at *m*/*z* 473.069 that yielded the fragment peak at *m*/*z* 311.041 (−162 Da, caffeoyl moiety) corresponding to the deprotonated caftaric acid. Similarly, metabolite **27** was recognized as dicaffeoylquinic acid due to the neutral loss of a caffeoyl moiety which yielded the fragment at *m*/*z* 353.087, the deprotonated form of caffeoylquinic acid. Compound **28** was recognized as coumaroyl-caffeoylquinic acid because of the neutral loss of the coumaroyl moiety (−146 Da) that produced the deprotonated caffeoylquinic acid at *m*/*z* 353.088. Similarly, metabolite **31** at *m*/*z* 529.135 was identified as caffeoyl-feruloylquinic acid due to the neutral loss of a caffeoyl moiety (−162 Da). The generated fragment at *m*/*z* 367.103 was the deprotonated form of feruloylquinic acid. This latter ion was found as precursor ion in peak **10**. The compound was tentatively recognized due to the typical daughter peaks of ferulic acid at *m*/*z* 193.049, 149.059, and 134.036. Also, compound **11** was identified as ferulic acid derivative. The parent ion at *m*/*z* 355.103 yielded the characteristic fragments of ferulic acid because of the loss of the hexoside (−162 Da). Similarly, compound **7** was tentatively identified as coumaroyl hexose due to the presence of the characteristic fragment ions of coumaric acid at *m*/*z* 163.039, 119. 049, and 93.033. Finally, the parent ion **6** at *m*/*z* 337.093 was assigned to coumaroylquinic acid because of the additional presence of the peak at *m*/*z* 191.055 related to the deprotonated form of quinic acid (loss of coumaroyl moiety, −146 Da).

Regarding the flavonoid class, derivatives of quercetin, kaempferol/luteolin, myricetin, and apigenin were present. Metabolites **14** and **15** were recognized as quercetin glucuronide and quercetin hexose because of the presence of quercetin fragment ions at *m*/*z* 301.035, 300.027, 271.024, and 255.029 generated from the loss of glucuronic acid and hexose moiety (−176 Da and −162 Da, respectively). The peak at *m*/*z* 609.146 (**19**) was assigned to rutin (quercetin rutinoside) due to the neutral loss of rutinoside moiety (−308 Da).

Kaempferol/luteolin derivatives (**12**, **20**, **21**, and **23**) were identified for the characteristic fragment peaks of the aglycone at *m*/*z* 285.040, 255.029, and 227.034. The parent ions at *m*/*z* 637.105 (**12**), 447.093 (**20**), 461.073 (**21**), and 593.151 (**23**) were recognized for the neutral loss of two consecutive glucuronic acid moieties (−176 Da), hexose (−162 Da), one glucuronic moiety (−176 Da), and rutinoside moiety (−308 Da), respectively.

Myricetin hexose was assigned to the parent ion **13** at *m*/*z* 479.083 because of the fragment peaks at *m*/*z* 317.030 (loss of the hexose, −162 Da) and 271.025.

Apigenin hexose and glucuronide (**25** and **26**) were identified for the aglycone characteristic daughter ions at *m*/*z* 269.045, 117.033 generated from the loss of and hexose (−162 Da) and a glucuronic moiety (−176 Da), respectively.

Finally, metabolites **40** and **41** were found in ROS and SAO biomasses and were identified as phenolic diterpenes, with a fragmentation pattern consistent with the literature [63]. Conversely, micropyrone (**39**) and arzanol (**42**) were tentatively identified in accordance with Kramberger et al. only in HEL biomass as they are among the most characteristic metabolites of this plant [49].

**Figure 2:**
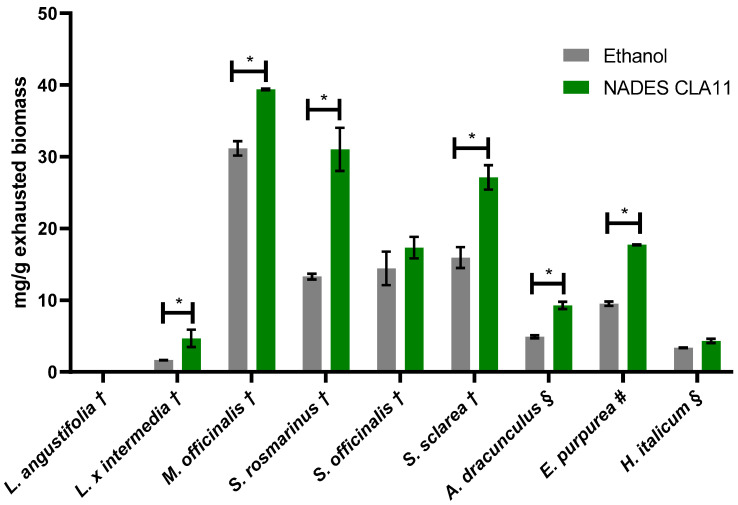
Amount of most characteristic polyphenols extracted with EtOH and NADES CLA11 in all biomasses. † rosmarinic acid; § dicaffeoylquinic acid; # chicoric acid. *, *p* < 0.05 according to Student’s *t*-test.

From the qualitative analysis, rosmarinic acid was the most abundant phenolic acid in all *Lamiaceae* biomasses. In addition, polyphenolic profiles of DRA and HEL [69,70,71,72] were mostly represented by dicaffeoylquinic acid as reported by other authors also in different species of the genus [73,74]. Thus, the quantification of these phenols in EtOH and CLA11 (Figure 2) extracts of all exhausted biomasses was performed by HPLC-DAD (the chromatograms are reported in the Supplementary Material).

The concentration of the target polyphenols was higher in all NADES extracts compared to the EtOH extracts, confirming the preliminary results on ECHI and MEL during the screening of the NADES formulations. In the case of LAV biomass, rosmarinic acid was not detected in both extracts. The detection of some polyphenols in this biomass via UHPLC-HRMS is ascribable to the high sensitivity of the analytical method. The lack of rosmarinic acid in *L. angustifolia* might be due to an extensive degradation of the phenolic acid during the steam distillation. The differences in the extractive performances of EtOH and NADES were not pronounced across all biomasses. However, the concentration of phenols in NADES extracts of LAI, ROS, SAS, DRA, and ECHI was significantly higher (*p* < 0.05) and nearly twice that of the EtOH extracts. In contrast, for SAO and HEL, the differences in polyphenol abundance were not significant, suggesting that NADES may not efficiently penetrate the plant material or solubilize the metabolites of interest. Similar results were achieved also by Jurić et al. on plants belonging to *Lamiaceae* family. Specifically, no differences in the yield of rosmarinic acid between EtOH 70% and NADES composed of ChCl with different HBD were observed in certain species, such as *O. basilicum* and *L. angustifolia*. The authors supposed that the type of solvent and the matrix-solvent interactions both play a central role in the extraction of specific compounds [28].

In general, the greater recovery suggested that the polarity of NADES closely aligns with that of the phenolic compounds in the plant biomasses, influencing their capability to dissolve the metabolites. The enhanced extraction capacity of NADES is attributed to the high number of hydrogen bonds between the polyphenols and the components of the mixtures. Therefore, the high solubility of these bioactive compounds within NADES is primarily due to the stabilization resulting from the intermolecular interactions they form with the NADES blends [13,75]. Moreover, Fan and co-workers proposed that the higher extractive performance of NADES on leaves of *Artemisia annua* L. is ascribable to the destruction of plant tissue as observed by scanning electron microscopy [76].

## 3. Materials and Methods

### 3.1. Chemicals and Sample Materials

Chicoric acid and C_8_–C_40_ n-alkanes mixture were provided from Sigma-Aldrich (Milan, Italy). Lactic and citric acid were purchased from Carlo Erba (Milan, Italy). Choline chloride (ChCl) was obtained from Tokyo Chemical Industry (Tokyo, Japan). Cynarine was obtained from LGC (North York, ON, Canada), while caftaric acid from Dr EHRENSTORFER (Augusta, Germany). Acetonitrile (ACN), acetic acid (HAc), formic acid, n-hexane (Hex), and ethanol (EtOH) were of LC–MS purity grade (Sigma-Aldrich, Milan, Italy).

The aerial parts of *Artemisia dracunculus* (ART), *Salvia rosmarinus* (ROS), and *Lavandula × intermedia* (LAI) were provided by Giardino delle Erbe “Rinaldi Ceroni”, Casola Valsenio (Ravenna, Italy) 6 JJF + 8 H, *Echinacea purpurea* (ECHI), *Helichrysum italicum* (HEL), *Lavandula angustifolia* (LAV), *Melissa officinalis* (MEL), *Salvia officinalis* (SAO), and *Salvia sclarea* (SAS) were provided by “La Bendessa” farm, Roncoscaglia, Sestola (Modena, Italy) 6 PRX + 59. All plants were hand-picked at full maturation during summer 2023.

### 3.2. Steam Distillation

The aerial parts of plants were harvested at the balsamic time and immediately steam distilled.

The steam distillation of fresh ART, ROS, and LAI was performed using an industrial apparatus equipped with a 250 L boiler (Albrigi Luigi s.r.l., Stallavena, VR, Italy) by Giardino delle Erbe “Rinaldi Ceroni”, Casola Valsenio (Ravenna, Italy) farm. The steam distillation of fresh ECHI, HEL, LAV, MEL, SAO, and SAS were performed using an industrial apparatus equipped with a 1500 L boiler (Albrigi Luigi s.r.l., Stallavena, VR, Italy) by the “Officine aromatiche del Frignano” with a 1500 L boiler (Albrigi). Briefly, aerial parts were steam distilled for 1 h and the EOs were collected in a Florentine flask and stored at room temperature in an amber glass bottle until the analysis. The oil-exhausted biomass of each plant was collected, air-dried, and stored at room temperature.

### 3.3. Chemical Characterization of the EOs

The obtained EOs were analyzed by GC to qualitatively and quantitatively determine their chemical composition.

#### 3.3.1. GC-MS Analysis

Analyses were carried out on a 7890 A gas chromatograph coupled with a 5975 C net-work mass spectrometer (GC-MS) (Agilent Technologies, Milan, Italy). Metabolites were separated on an Agilent Technologies HP-5 MS cross-linked poly-5% diphenyl–95% dimethyl polysiloxane (30 m × 0.25 mm i.d., 0.25 μm film thickness) capillary column. The column temperature was initially set at 45 °C, then increased at a rate of 2 °C/min up to 100 °C, then raised to 250 °C at a rate of 5 °C/min and maintained for 5 min. The EOs were diluted 1:20 (*v*/*v*) with n-hexane and 0.1 μL were injected, operating in split mode with a split ratio 1:20. Helium was used as the carrier gas and set at the flow rate of 0.7 mL/min. The injector, transfer line, and ion-source temperature were 250, 280, and 230 °C, respectively. MS detection was achieved with electron ionization at 70 eV, operating in the full-scan acquisition mode in the *m*/*z* range 40–400. A comparison of the MS fragmentation pattern of the target analytes with those of pure components was carried out using the NIST mass-spectral database.

#### 3.3.2. GC-FID Analysis

Chromatographic characterization of EOs was performed on a 7820 gas chromatograph (Agilent Technologies, Milan, Italy) coupled with FID. EOs and the mixture of aliphatic hydrocarbons (C_8_–C_40_) were diluted 1:20 (*v*/*v*) with Hex before GC-FID analysis and 1 μL was injected. Helium was employed as carrier gas at the flow rate of 1 mL/min. The injector and detector temperatures were set at 250 and 300 °C, respectively. The components of the EOs were separated on an Agilent Technologies HP-5 crosslinked poly-5% diphenyl–95% dimethylsiloxane (30 m *×* 0.32 mm i.d., 0.25 μm film thickness) capillary column. The column temperature was initially set at 45 °C, then increased at a rate of 2 °C/min up to 100 °C, then raised to 250 °C at a rate of 5 °C/min, and finally held for 5 min. The injection was performed in split mode with a split ratio 1:20.

Metabolites were identified by comparing the retention times of the peaks with those of authentic reference standards run under the same conditions. Additionally, the identification was achieved by comparing the LRIs relative to C_8_–C_40_ n-alkanes obtained on the HP-5 column under the above-mentioned conditions with the literature. Peak enrichment by co-injection with authentic reference compounds was also carried out. The relative percentage of individual metabolites was expressed as the peak area percentage relative to the total peak area in the chromatogram. Semi-quantitative data were calculated as the mean of two analyses.

The data acquisition and processing were performed using the OpenLab CDS C.01.04 (Agilent Technologies, Santa Clara, CA, USA) software.

### 3.4. Preparation of NADES Mixtures

ChCl was selected as the HBA while LA and CA were tested as HBD. The preparation of the NADES formulations was performed according to Bakirtzi et al. [32]. Briefly, a fixed amount of ChCl was mixed with the HBDs at different molar ratios as reported in Table 3. The mixtures were heated under stirring at 50 °C until a transparent and homogeneous liquid was obtained. Afterwards, 20% (*v*/*v*) of water was added to the mixtures to reduce the viscosity of the NADES.

### 3.5. Oil-Exhausted Biomass Extraction

The exhausted biomasses obtained from the steam distillation were grounded and extracted with both EtOH, as conventional solvent, and different NADES mixtures.

#### 3.5.1. Ethanolic Extraction

Approximately 250 mg of each sample was extracted by ultrasonication with 40 mL of 70% EtOH at room temperature for 15 min. The sample was then centrifuged at 3200 rpm for 5 min, and the supernatant was filtered through paper. The biomass was further extracted with 40 mL, followed by 20 mL of the same solvent, under the same conditions. Finally, the filtrates were combined in a 100 mL volumetric flask, and the solution was stored at −4 °C until analysis. All extractions were performed in triplicate.

#### 3.5.2. NADES Extraction

The NADES extraction was carried out according to Bakirtzi et al. [32]. Briefly, 500 mg of each sample was extracted with 25 mL of NADES under ultrasonication for 90 min at 80 °C. The sample was then vacuum-filtered through paper, and the filtrate was diluted with Milli-Q water in a 50 mL volumetric flask.

### 3.6. Characterization of Biomass Extracts

The EtOH extracts and the optimized NADES extracts of ART, ECHI, HEL, LAV, LAI, MEL, SAO, ROS, and SAS were analyzed by UHPLC-HRMS to determine the polyphenolic composition. Subsequently, the most characteristic compounds were quantified using HPLC-DAD.

#### 3.6.1. UHPLC-HRMS Analysis

The analyses were performed on a Thermo Scientific (Waltham, MA, USA) UHPLC Ultimate 3000, equipped with a binary pump, a vacuum degasser, a thermostated autosampler, a thermostated column compartment, and a Q-Exactive Orbitrap mass spectrometer, with a heated electrospray ionization (HESI) source. An Ascentis Express C_18_ column (100 mm × 2.1 mm I.D., 3 µm, Supelco, Milan, Italy) was used. The mobile phase was composed of (A) 0.1% HCOOH in water and (B) 0.1% HCOOH in ACN, and the gradient elution was set as follows: 0–2 min, 2% B; 2–20 min, 35% B; 20–25 min, 98% B; 25–35 min, 2%. The flow rate was set at 0.4 mL/min and the injection volume was 10 μL. The column temperature was 25 °C.

MS acquisition was performed in negative ionization mode. The source parameters were set as follows: sheath gas (N_2_) 45, auxiliary gas (N_2_) 25, sweep gas (N_2_) 2, auxiliary gas temperature 290 °C, and electrospray voltage 3.80 kV (+) and 3.40 kV (−). A data-dependent acquisition strategy was used to acquire both Full MS and higher energy collisional dissociation (HCD) fragmentation spectra. Mass analyzer acquisition parameters were set as follows: Full MS scan range 100 < *m*/*z* < 100 at 35,000 full width half maximum (FWHM) resolving power with an automatic gain control (AGC) target set at 1 × 10^6^ ions with 200 ms maximum injection time; top 2 HCD fragmentation spectra of most abundant precursor ions were acquired at 17,500 FWHM resolving power using an isolation window of 1.0 *m*/*z* and stepped normalized collision energy (NCE) at 20, 30, and 50.

#### 3.6.2. HPLC-DAD Analysis

Chromatographic analyses were performed using the Agilent 1260 Infinity II instrument (Agilent Technologies), which includes a quaternary pump (Quaternary Pump 1260, Agilent Technologies), an autosampler (Vialsampler 1260, Agilent Technologies), and a UV/DAD detector (Diode Array WR 1260, Agilent Technologies). Chromatograms were recorded and analyzed using the Agilent Open Lab CDS Version 2.6 software (Agilent Technologies). Chromatographic separation was conducted using two previously developed and validated methods, one for the ECHI samples and the other one for the remaining samples. For both methods, the flow rare was set at 1 mL/min and the injection volume was 10 µL. Before injection, all samples were filtered through a 0.2 µm PTFE (polytetrafluoroethylene) filter and then poured into the vials. All the analyses were carried out in duplicate.

For the ECHI samples, the mobile phase was composed of (A) 0.1% HCOOH in water and (B) ACN, and the gradient elution was set as follows: 0 min, 15% B; 0–10 min, 30% B; 10–18 min, 65% B; 18–25, 80% B; 25–30 min, 90% B. The total run time was 32 min and the equilibration time was 5 min.

For all the other samples, the mobile phase was composed of (A) 0.3% HAc in water and (B) ACN, and the gradient was set as follows: 0 min, 17% B; 0–35 min, 23% B; 35–52 min, 49% B.

### 3.7. Statistical Analyses

Significant differences within groups were determined at *p* < 0.05 using one-way analysis of variance (ANOVA) followed by Tukey’s post hoc test and Student’s *t*-test via GraphPad Prism 8.4.3 (GraphPad Software, San Diego, CA, USA).

## 4. Conclusions

This study has provided an overview of the method to recover these biomasses using natural deep eutectic mixture solvents, known as NADES. Through advanced analytical techniques like HPLC-DAD, and UHPLC-HRMS, the research has shown that NADES offer a higher extraction capacity compared to conventional organic solvents, such as EtOH. The preliminary screening on different NADES formulations highlighted that viscosity might play a central role in the recovery of the most concentrated phenolic acids. NADES composed of choline chloride in combination with CA were demonstrated to be less efficient in the preliminary screening probably due to the greater viscosity of these eutectic solvents. Conversely, the NADES composed of ChCl and LA at the molar ratio of 1:1 recovered the most concentrated phenolic acid in all plants, in most cases significantly, and therefore was selected as the best formulation for further studies. From the UHPLC-HRMS analysis, this NADES formulation was demonstrated to be capable of extracting a higher number of compounds in all exhausted biomasses.

The study proved that NADES can be a suitable solution for efficiently valorizing the production chain of EOs and making it more sustainable both in terms of polyphenol recovery and impact on the environment. Indeed, NADES exhibit several environmentally friendly characteristics that enhance their sustainability profile, one of them being their biodegradability. Additionally, unlike EtOH, NADES are biocompatible and non-toxic, making them safe for biological systems and minimizing risks to human health, animal health, and the environment. Moreover, to the best of our knowledge, this is the first study focused on the recovery of polyphenols from the by-product of steam distillation of aromatic plants using NADES.

Looking ahead, future research should continue to explore NADES in the context of sustainability by also evaluating the recycling of eutectic solvents with the of making the process more and more sustainable. Further investigations should focus on their environmental impact throughout their lifecycle and their potential to support circular economy principles. Additionally, it is essential to evaluate potential formulations containing NADES extracts from aromatic plant biomasses for applications in the pharmaceutical, nutraceutical, and cosmetic industries.

## Figures and Tables

**Table 2:** Chemical composition of waste distillation biomasses extracted by EtOH 70% or ChCl and lactic acid at molar ratio 1:1 (CLA11) by UHPLC-HRMS.

No	Rt (min)	Molecule	(M-H) (*m/z*)	Error (ppm)	Fragments (*m/z*)	Formula	Molecular Weight (g/mol)		Extract		Reference
									EtOH	NADES	
1	2.52	Danshensu	197.0449	0.70	179.0342, 135.0441, 123.0440, 72.9918	C9H10O5	198.052824	LAI LAV MEL ROS SAO SAS ART ECHI HEL	+ + + + + + − − −	+ + + + + + − − −	[48]
2	2.6	Protocatechuic acid hexose	315.0723	−2.19	153.0184, 152.0106, 109.0282, 108.0205	C13H16O9	316.079435	LAI LAV MEL ROS SAO SAS ART ECHI HEL	+ + + + + + + + −	+ + + + + + + + −	[49]
3	3.16	Caftaric acid	311.0408	−1.58	179.0342, 149.0082, 135.0441	C13H12O9	312.048135	LAI LAV MEL ROS SAO SAS ART ECHI HEL	+ − − − − − − + −	+ − − − − − − + −	[50]
4	3.79	Caffeoylquinic acid isomer I	353.0876	−0.96	191.0554, 179.0342, 135.0441	C16H18O9	354.095085	LAI LAV MEL ROS SAO SAS ART ECHI HEL	− − − + + − + − +	− − − + + − + − +	[49]
5	4.34	Caffeoyl hexose isomer I	341.0878	−1.58	179.0342, 161.0235, 135.0442	C15H18O9	342.095085	LAI LAV MEL ROS SAO SAS ART ECHI HEL	− − + + + − − − −	+ − + + + − + + −	[51]
6	4.79	Coumaroyl quinic acid	337.0931396	−2.36	191.0555, 163.0392, 119.0490, 93.0333	C16H18O8	338.100170	LAI LAV MEL ROS SAO SAS ART ECHI HEL	− − − − − − + − +	− − − − − − + − +	[52]
7	5.01	Coumaroyl hexose	325.0929	−1.71	183.0114, 163.0392, 119.0492, 93.0333	C15H18O8	326.100170	LAI LAV MEL ROS SAO SAS ART ECHI HEL	+ + + + − − + − +	+ + + + + − + − +	[10]
8	5.35	Caffeoyl hexose isomer II	341.0877	−1.29	179.0342, 161.0235, 135.0441	C15H18O9	342.095085	LAI LAV MEL ROS SAO SAS ART ECHI HEL	− − + + + − − − −	+ − + + + − + + −	[51]
9	5.66	Caffeoylquinic acid isomer II	353.0876	−0.96	191.0554, 179.0342, 135.0441	C16H18O9	354.095085	LAI LAV MEL ROS SAO SAS ART ECHI HEL	− − − + + − + − +	− − − + + − + − +	[49]
10	6.42	Feruloylquinic acid	367.1031	−0.52	193.0499, 149.0598, 134.0363	C17H20O9	368.110735	LAI LAV MEL ROS SAO SAS ART ECHI HEL	− − − − − − + − −	− − − − − − + − −	[53]
11	6.48	Feruloyl hexose	355.1035	−1.66	193.0499, 149.0598, 134.0362, 119.0339	C16H20O9	356.110735	LAI LAV MEL ROS SAO SAS ART ECHI HEL	+ + − + + − + − −	+ + − + + − + − −	[10]
12	8.92	Luteolin/kaempferol diglucuronide	637.1053	−1.89	461.0721, 285.0405, 255.0298, 227.0349	0	638.111920	LAI LAV MEL ROS SAO SAS ART ECHI HEL	− − − − + − − − −	+ + − − + − − − −	[10]
13	9.32	Myricetin hexose	479.0837	−2.36	317.0305, 271.0250	C21H20O13	480.090395	LAI LAV MEL ROS SAO SAS ART ECHI HEL	− − − − − − − − +	− − − − − − − − +	[53]
14	9.63	Quercetin glucuronide	477.0680	2.63	301.0359, 300.0286, 271.02567, 255.0287	C21H18O13	478.074745	LAI LAV MEL ROS SAO SAS ART ECHI HEL	− − − − + − − − −	− − − − + − − − −	[54]
15	9.68	Quercetin hexose	463.0888	−2.47	301.0359, 300.02719, 271.02567, 255.02921	C21H20O12	464.095480	LAI LAV MEL ROS SAO SAS ART ECHI HEL	− − − + − − − − +	− − − + − − − − +	[49]
16	9.78	Yunnaneic acid D	539.1196	−1.20	359.0777, 297.0771, 197.0452, 179.0342, 161.0236, 135.0441	C27H24O12	540.126780	LAI LAV MEL ROS SAO SAS ART ECHI HEL	+ − + + − − − − −	+ + + + − − − − −	[55]
17	9.86	Chicoric acid	473.0692	5.93	311.0411, 293.0308, 179.0343, 149.0082	C22H18O12	474.079830	LAI LAV MEL ROS SAO SAS ART ECHI HEL	− − + − − − − + −	− − + − − − − + −	[56]
18	10.02	Yunnaneic acid F	597.1254	−1.62	311.0930, 197.0449, 179.0342, 135.0441	C29H26O14	598.132260	LAI LAV MEL ROS SAO SAS ART ECHI HEL	− − − + + − − − −	+ − − + + − − − −	[48]
19	10.51	Rutin	609.1468	−2.03	300.0278, 271.0251, 255.0301	C27H30O16	610.153390	LAI LAV MEL ROS SAO SAS ART ECHI HEL	− − − + − − + + −	+ − − + − + + + −	[57]
20	10.74	Luteolin/kaempferol hexose	447.0939	−2.60	285.0405, 255.0298, 227.0349	C21H20O15	448.100564	LAI LAV MEL ROS SAO SAS ART ECHI HEL	− − − − + + − − +	− − − − + + − − +	[58]
21	10.90	Luteolin/kaempferol glucuronide	461.0731	−2.37	285.0405, 255.0298, 227.0349	C21H18O12	462.079830	LAI LAV MEL ROS SAO SAS ART ECHI HEL	+ + − + + + − − −	+ + + + + + − − −	[10]
22	11.06	Rosmarinic acid hexose	521.1304	−1.69	359.0766, 197.0449, 179.0342, 161.0236, 135.0440	0	521.129520	LAI LAV MEL ROS SAO SAS ART ECHI HEL	− − + + + − − − −	− − + + + − − − −	[57,59,60]
23	11.81	Luteolin/kaempferol rutinose	593.15184	1.09	285.04041, 255.02995, 227.03470,	C27H30O15	594.15912	LAI LAV MEL ROS SAO SAS ART ECHI HEL	− − − − + − + − +	− − − + + − + − +	[61]
24	11.92	Rosmarinic acid	359.0776	−2.52	197.0449, 179.0344, 161.0235, 135.0441	C18H16O8	360.084520	LAI LAV MEL ROS SAO SAS ART ECHI HEL	+ + + + + + − − −	+ + + + + + + − −	[62]
25	12.09	Apigenin hexose	431.0988	−2.37	269.0454, 117.0332	C21H20O10	432.105649	LAI LAV MEL ROS SAO SAS ART ECHI HEL	+ + − − − + − − −	+ + + − − + − − −	[58]
26	12.30	Apigenin glucuronide	445.0782	−2.49	269.0457, 117.0334	C21H18O11	446.084915	LAI LAV MEL ROS SAO SAS ART ECHI HEL	− − − − − + − − −	− − − − + + − − −	[48]
27	12.43	Dicaffeoyl quinic acid	515.14105	0.73	353.08780, 191.05553, 179.03413, 135.04416	C25H24O12	516.147900	LAI LAV MEL ROS SAO SAS ART ECHI HEL	− − − − − − + − +	− − − − − − + − +	[61]
28	12.72	Coumaroyl-caffeoylquinic acid	499.1250	−1.92	353.0886, 337.0932, 191.0555, 173.0447	C25H24O11	500.131865	LAI LAV MEL ROS SAO SAS ART ECHI HEL	− − − − − − + − −	− − − − − − + − −	[52]
29	12.86	Methylluteolin-O-glucuronide (Kaempferide glucuronide)	475.0888	−2.41	299.0561, 284.0327	C22H20O12	476.095480	LAI LAV MEL ROS SAO SAS ART ECHI HEL	− − − + + + − − −	− − − + + + − − −	[63,64]
30	12.89	Salvianolic acid K	555.1122	3.01	359.0778, 179.0341, 161.0235, 135.0441	C27H24O13	556.121695	LAI LAV MEL ROS SAO SAS ART ECHI HEL	− − + − + + − − −	+ − + + + + − − −	[63]
31	13.28	Caffeoyl-feruloylquinic acid	529.1356	−1.88	367.1036, 179.0340, 161.0236, 135.0442	C26H26O12	530.142430	LAI LAV MEL ROS SAO SAS ART ECHI HEL	− − − − − − + − −	− − − − − − + − −	[52]
32	13.47	Salvianolic acid H (lithospermic acid)	537.1042	−1.67	359.0777. 295.0613. 179.0334. 161.0234. 135.0441	C27H22O12	538.111130	LAI LAV MEL ROS SAO SAS ART ECHI HEL	− − − − + + − − −	− − + + + + − − −	[65]
33	15.30	Salvianolic acid A	493.1140	−1.06	295.0613, 197.0451, 179.0343, 135.0442	C26H22O10	494.121300	LAI LAV MEL ROS SAO SAS ART ECHI HEL	+ − − + + − − − −	+ − + + + + − − −	[48]
34	15.33	Sagecoumarin isomer I	535.0882	−1.02	359.0775, 197.0443, 179.0341, 177.0186, 161.0234, 135.0443	C27H20O12	536.095480	LAI LAV MEL ROS SAO SAS ART ECHI HEL	− − + − + − − − −	− − + − + + − − −	[66]
35	16.03	Sagecoumarin isomer II	535.0882	−1.02	359.0775, 197.0443, 179.0341, 177.0186, 161.0234, 135.0443	C27H20O12	536.095480	LAI LAV MEL ROS SAO SAS ART ECHI HEL	− − + − + − − − −	− − + − + + − − −	[63]
36	16.21	Salvianolic acid C	491.0989	−2.14	311.0566, 265.05, 179.0360, 135.0442	C26 H20 O10	492.105649	LAI LAV MEL ROS SAO SAS ART ECHI HEL	− − + + − − − − −	+ − + + − + − − −	[48]
37	12.13	Salvianolic acid B	717.1473	−2.42	519.0939, 339.051 161.023, 135.0444	C36H30O16	718.153390	LAI LAV MEL ROS SAO SAS ART ECHI HEL	+ − + − + + − − −	+ − + − + + − − −	[55]
38	12.31	Sagerinic acid	719.1624	1.64	359.0776, 197.0449, 179.0342, 161.0235	C36H32O16	720.169040	LAI LAV MEL ROS SAO SAS ART ECHI HEL	+ − + + + + − − −	+ − + + + + − − −	[63]
39	15.83	Micropyrone	251.1289	2.39	207.1385, 151.1118, 113.0960	C14H20O4	252.136160	LAI LAV MEL ROS SAO SAS ART ECHI HEL	− − − − − − − − +	− − − − − − − − +	[49]
40	22.70	Rosmadial (safficinolide)	343.1552	−1.75	299.16509	C20 H24 O5	344.162375	LAI LAV MEL ROS SAO SAS ART ECHI HEL	− − − + + − − − −	− − − + + − − − −	[67]
41	22.88	Carnosol	329.1758	−1.60	285.1862	C20H26O4	330.183110	LAI LAV MEL ROS SAO SAS ART ECHI HEL	− − − + + − − − −	− − − + + − + − −	[62,68]
42	23.52	Arzanol	401.1609	−2.17	247.0976, 235.0974, 191.1071, 166.0263, 153.0548, 109.0647	C22H26O7	402.167855	LAI LAV MEL ROS SAO SAS ART ECHI HEL	− − − − − − − − +	− − − − − − − − +	[49]

**Table 3:** NADES formulations prepared.

**Name**	**Eutectic Mixture**	**Molar Ratio**
CCA11	Choline chloride: Citric acid	1:1
CCA12	Choline chloride: Citric acid	1:2
CLA11	Choline chloride: Lactic acid	1:1
CLA12	Choline chloride: Lactic acid	1:2

## Data Availability

The data presented in this study are available on request from the corresponding author.

## Supplementary Materials

The following supporting information can be downloaded at: https://www.mdpi.com/article/10.3390/molecules30051120/s1, Figure S1. HPLC/DAD chromatogram of DRA ethanol extract; Figure S2. HPLC/DAD chromatogram of ECHI ethanol extract; Figure S3. HPLC/DAD chromatogram of HEL ethanol extract; Figure S4: HPLC/DAD chromatogram of LAI ethanol extract; Figure S5: HPLC/DAD chromatogram of LAV ethanol extract; Figure S6. HPLC/DAD chromatogram of MEL ethanol extract; Figure S7. HPLC/DAD chromatogram of ROS ethanol extract; Figure S8. HPLC/DAD chromatogram of SAO ethanol extract; Figure S9. HPLC/DAD chromatogram of SAS ethanol extract; Figure S10. HPLC/DAD chromatogram of DRA NADES extract; Figure S11. HPLC/DAD chromatogram of ECHI NADES extract; Figure S12. HPLC/DAD chromatogram of HEL NADES extract; Figure S13. HPLC/DAD chromatogram of LAI NADES extract; Figure S14. HPLC/DAD chromatogram of LAV NADES extract; Figure S15. HPLC/DAD chromatogram of MEL NADES extract; Figure S16. HPLC/DAD chromatogram of ROS NADES extract; Figure S17. HPLC/DAD chromatogram of SAO NADES extract; Figure S18. HPLC/DAD chromatogram of SAS NADES extract.

## Abbreviations

The following abbreviations are used in this manuscript:

EO	Essential oil
NADES	Natural deep eutectic solvents
HBA	Hydrogen bond acceptor
HBD	Hydrogen bond donator
ART	*Artemisia dracunculus*
ECHI	*Echinacea purpurea*
HEL	*Helichrysum italicum*
LAV	*Lavandula angustifolia*
LAI	*Lavandula × intermedia*
MEL	*Melissa officinalis*
SAO	*Salvia officinalis*
ROS	*Salvia rosmarinus*
SAS	*Salvia sclarea*
GC	Gas chromatography
UHPLC-HRMS	Ultrahigh-Performance Liquid Chromatography–High-Resolution Mass Spectrometry
HPLC-DAD	High-Performance Liquid Chromatography with Diode Array Detection
LRI	Linear retention index
EtOH	Ethanol
CA	Citric acid
LA	Lactic acid
Hex	Hexane
ChCl	Choline chloride
