# Supplementary material for: Recovery of Bioactive Compounds from the Biomass of Aromatic Plants After Distillation Using NADES: A Sustainable Alternative Extraction Method
Source: Molecules. 2025 Feb 28;30(5):1120. doi: 10.3390/molecules30051120 (PMC11901988; doi:10.3390/molecules30051120)
Supplement: Supplementary file 1 [file molecules-30-01120-s001.zip › molecules-3479316-supplementary.pdf]

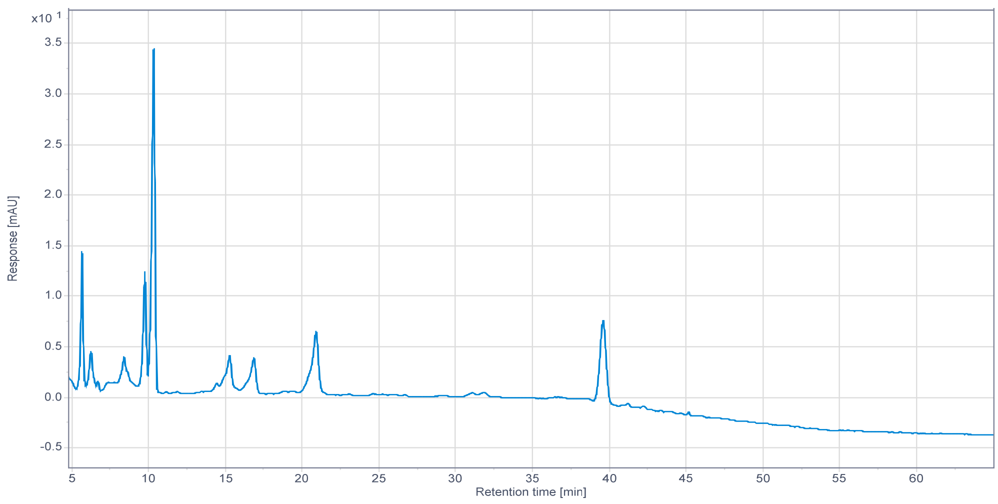

Figure S1. HPLC/DAD chromatogram of DRA ethanol extract

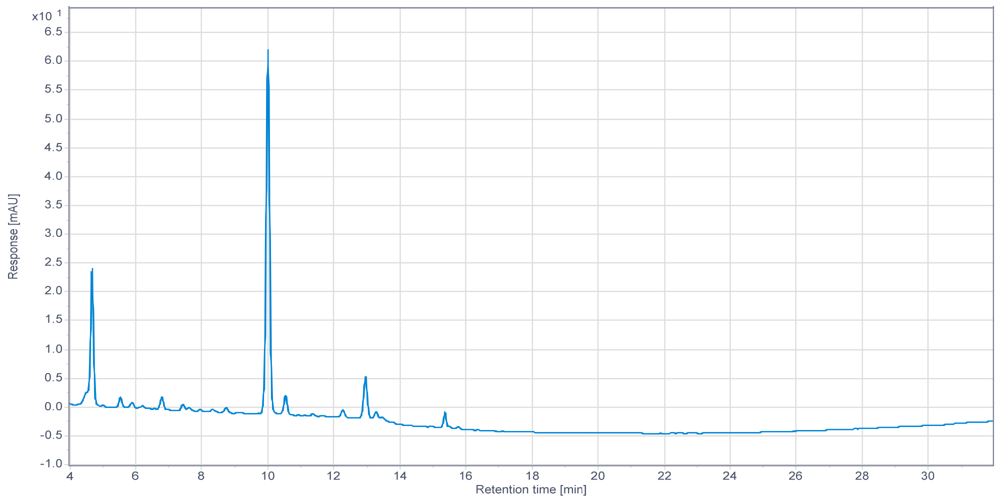

Figure S2. HPLC/DAD chromatogram of ECHI ethanol extract

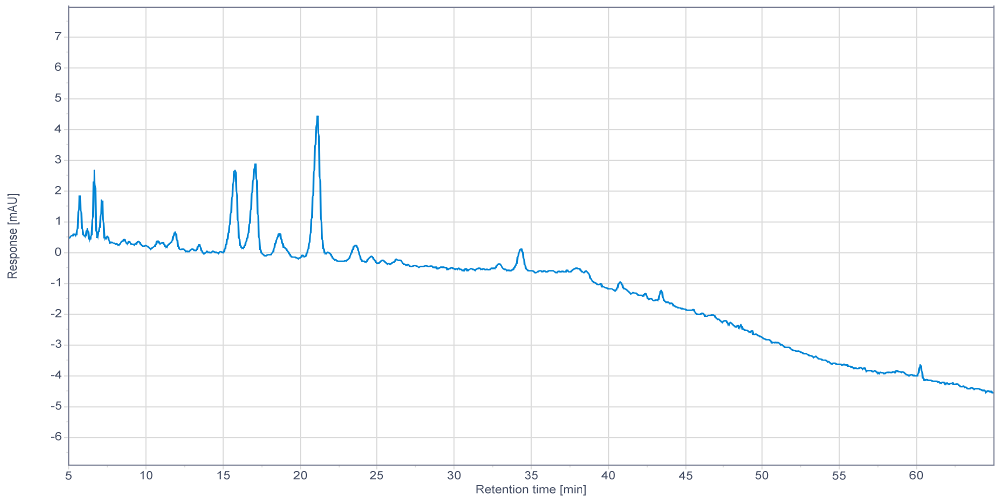

Figure S3. HPLC/DAD chromatogram of HEL ethanol extract

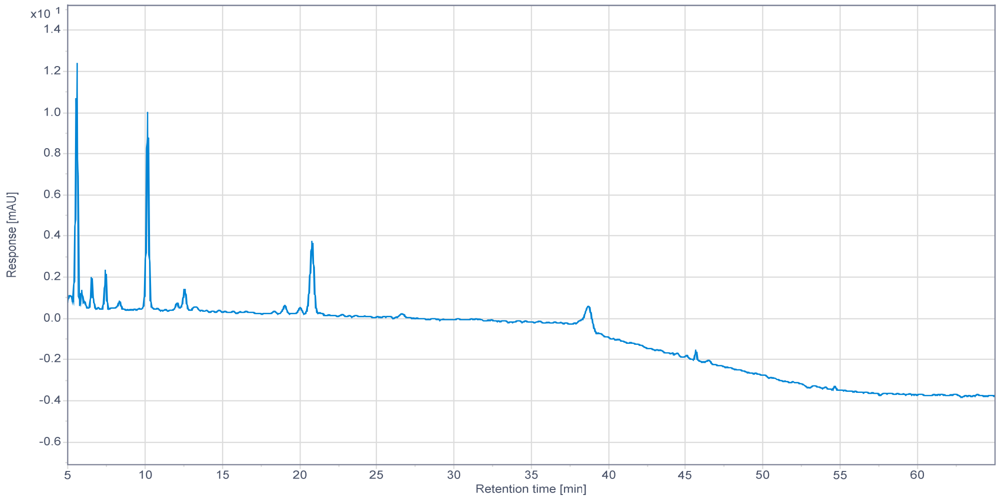

Figure S4: HPLC/DAD chromatogram of LAI ethanol extract

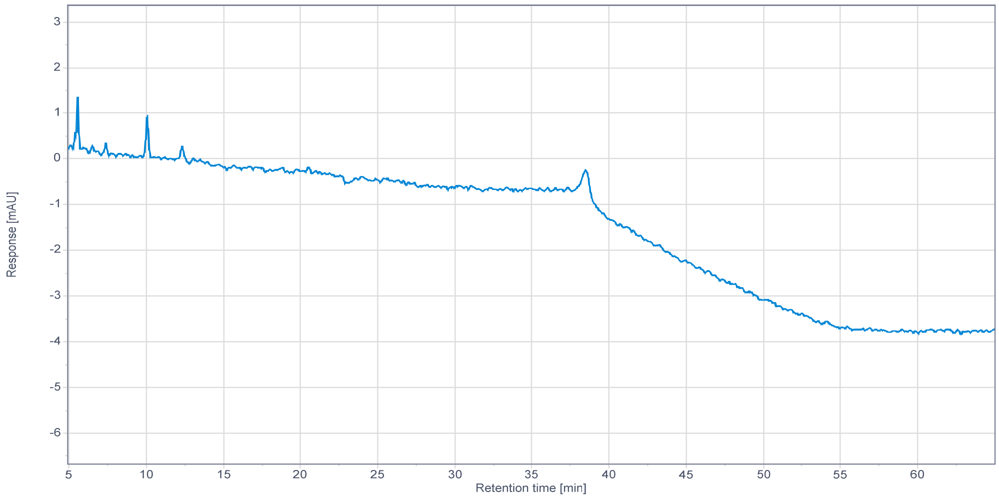

Figure S5: HPLC/DAD chromatogram of LAV ethanol extract

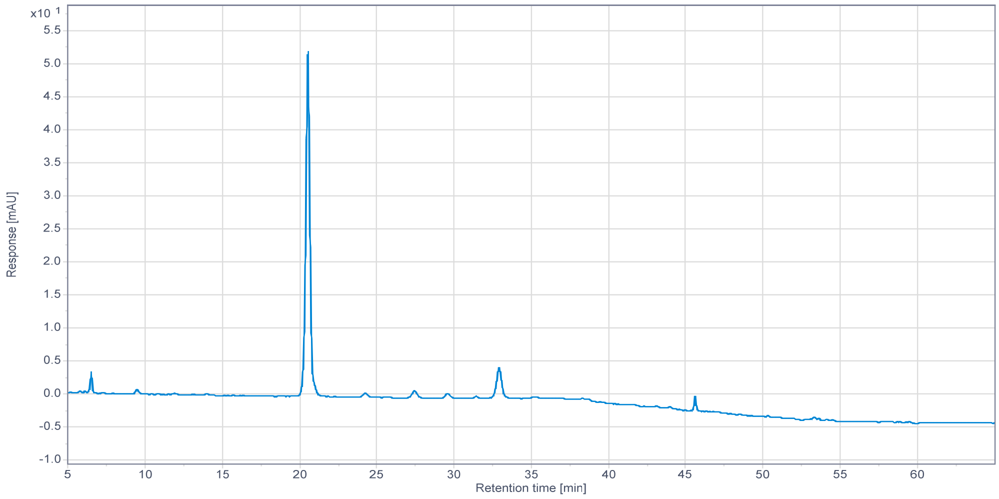

Figure S6. HPLC/DAD chromatogram of MEL ethanol extract

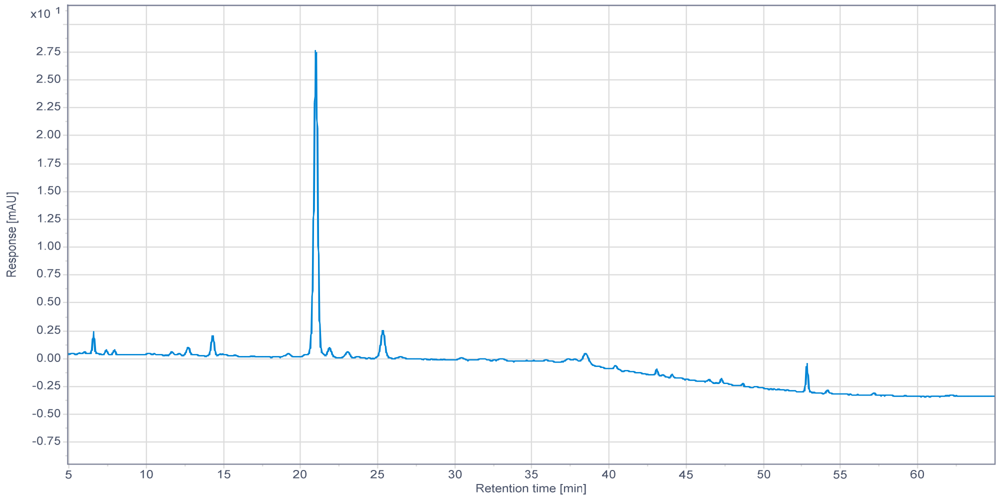

Figure S7. HPLC/DAD chromatogram of ROS ethanol extract

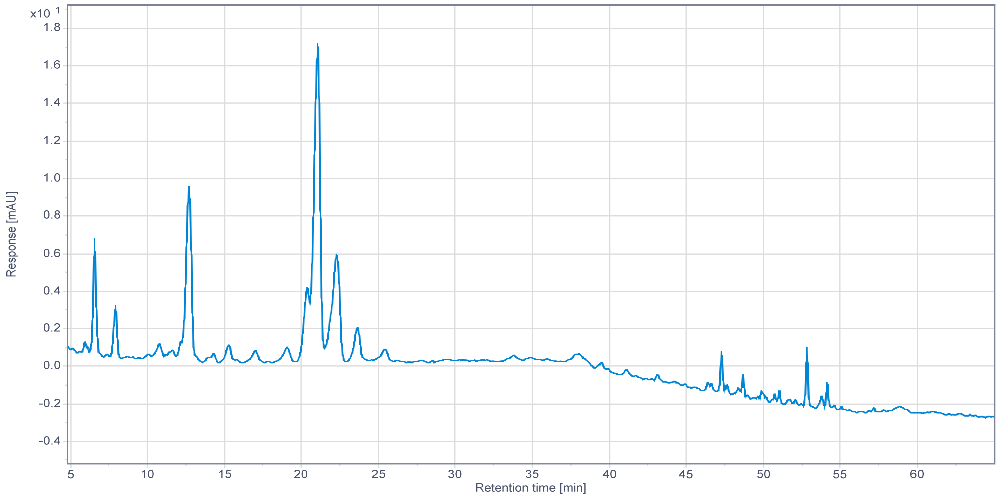

Figure S8. HPLC/DAD chromatogram of SAO ethanol extract

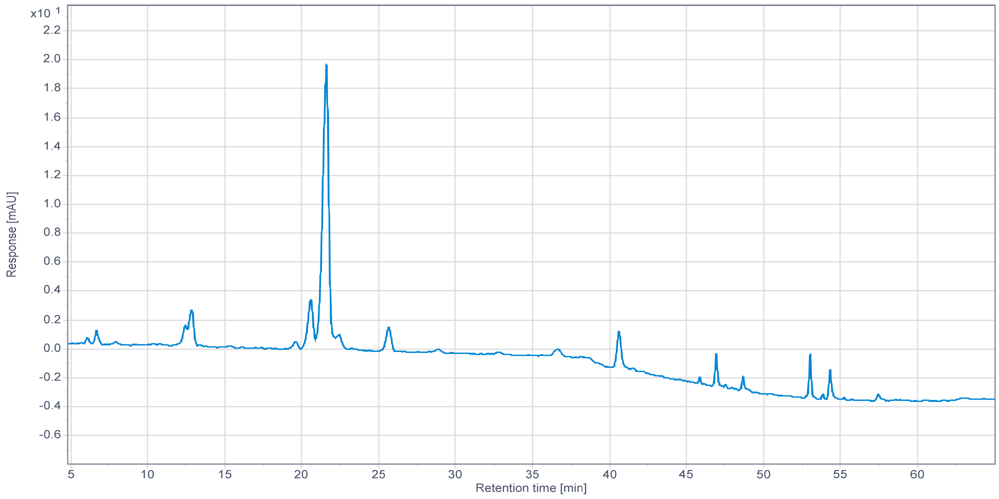

Figure S9. HPLC/DAD chromatogram of SAS ethanol extract

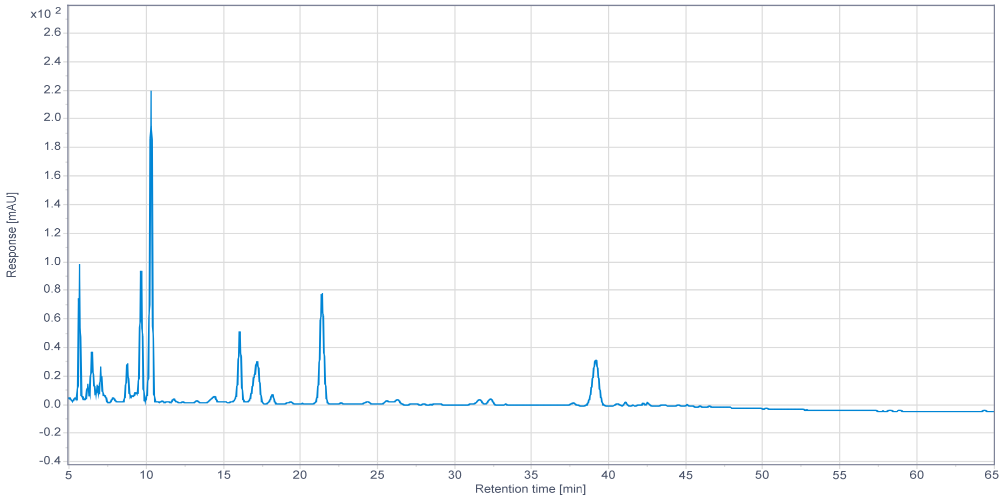

Figure S10. HPLC/DAD chromatogram of DRA NADES extract

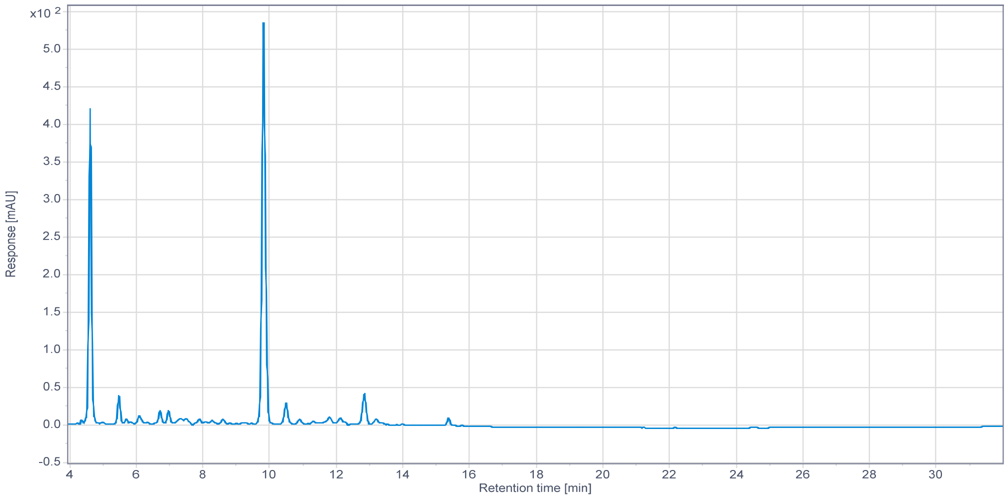

Figure S11. HPLC/DAD chromatogram of ECHI NADES extract

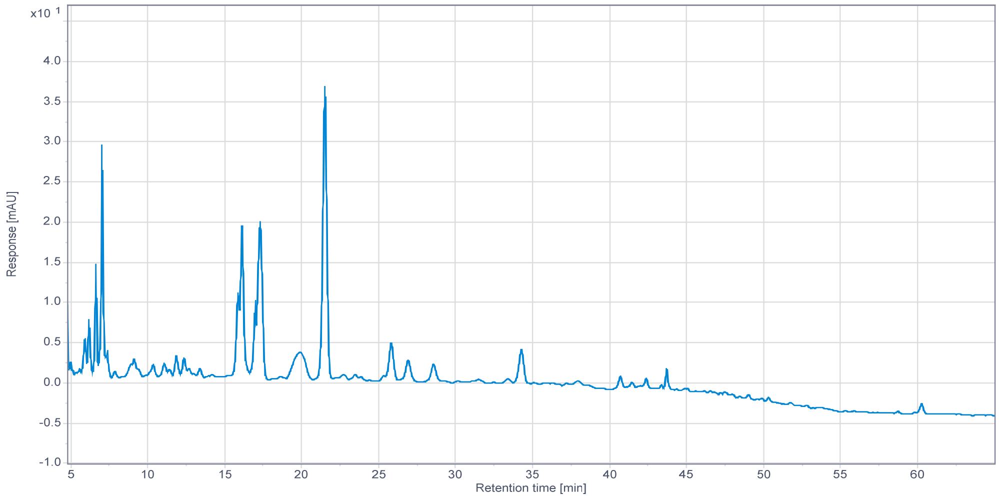

Figure S12. HPLC/DAD chromatogram of HEL NADES extract

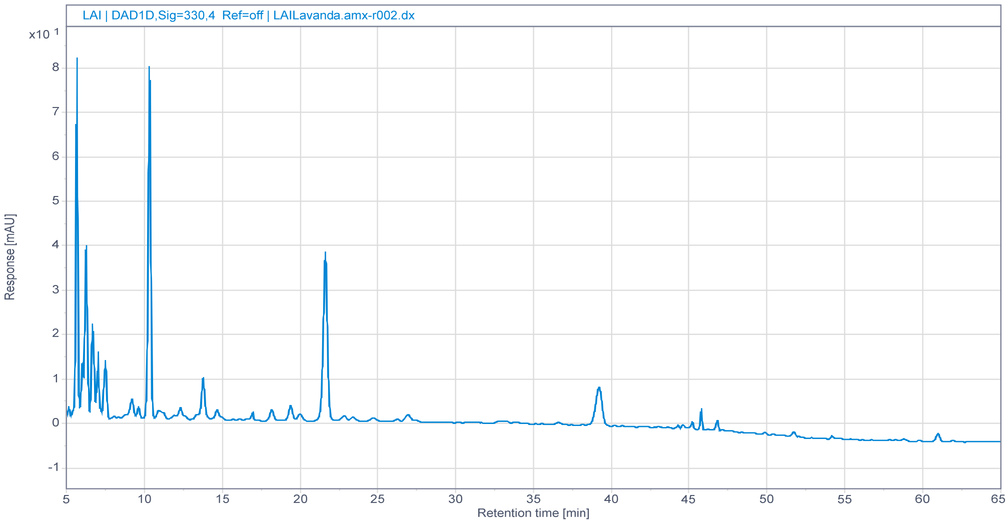

Figure S13. HPLC/DAD chromatogram of LAI NADES extract

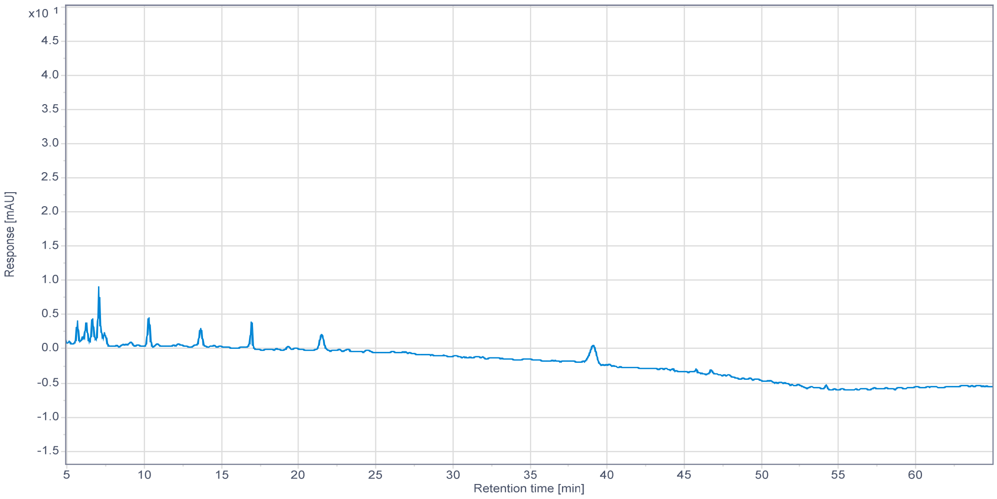

Figure S14. HPLC/DAD chromatogram of LAV NADES extract

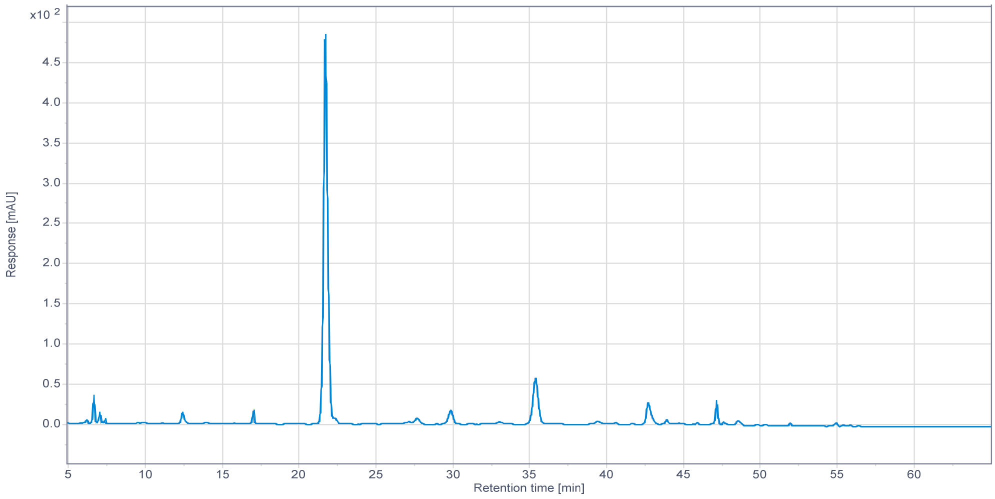

Figure S15. HPLC/DAD chromatogram of MEL NADES extract

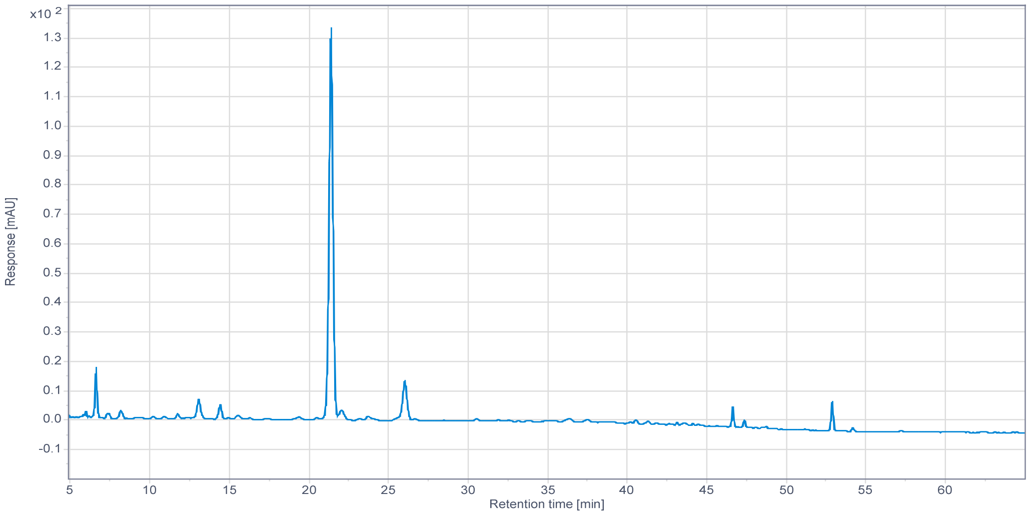

Figure S16. HPLC/DAD chromatogram of ROS NADES extract

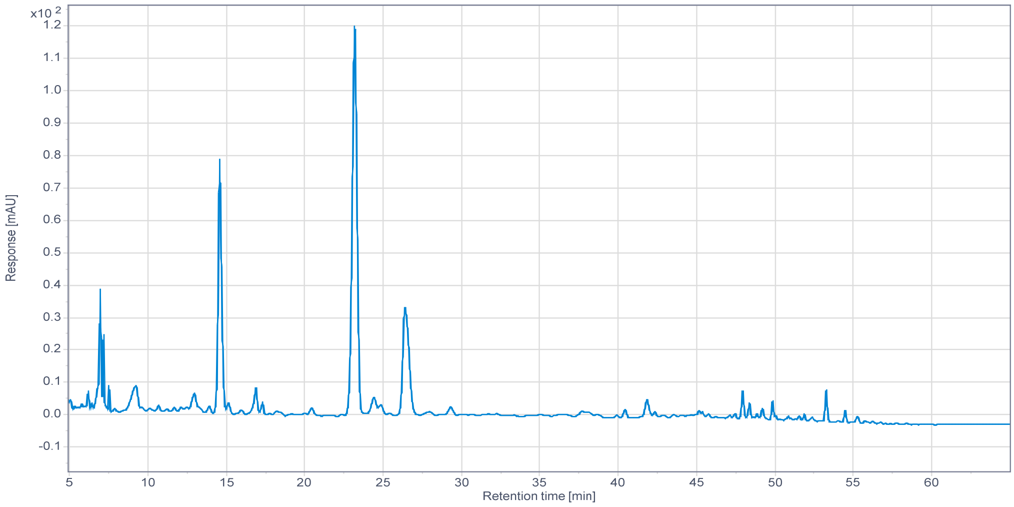

Figure S17. HPLC/DAD chromatogram of SAO NADES extract

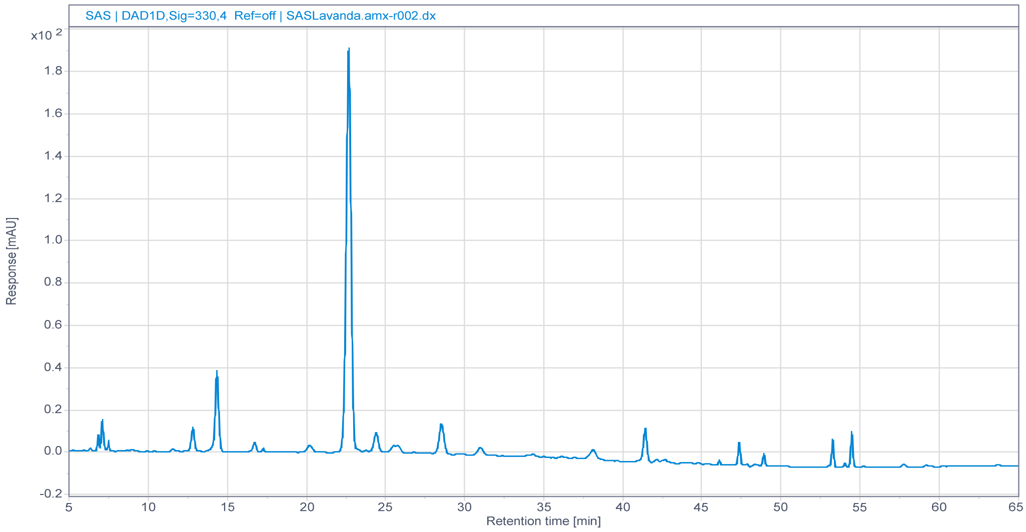

Figure S18. HPLC/DAD chromatogram of SAS NADES extract
